# Plasma amino acid profile associated with fatty liver disease and co-occurrence of metabolic risk factors

**DOI:** 10.1038/s41598-017-14974-w

**Published:** 2017-11-03

**Authors:** Minoru Yamakado, Takayuki Tanaka, Kenji Nagao, Akira Imaizumi, Michiharu Komatsu, Takashi Daimon, Hiroshi Miyano, Mizuki Tani, Akiko Toda, Hiroshi Yamamoto, Katsuhisa Horimoto, Yuko Ishizaka

**Affiliations:** 1 Center for Multiphasic Health Testing and Services, Mitsui Memorial Hospital, 1 Kanda, Izumicho, Chiyoda-ku, Tokyo, 101-8643 Japan; 2 Institute for Innovation, Ajinomoto Co., Inc., 1-1 Suzuki-cho, Kawasaki-ku, Kawasaki, 210-8681 Japan; 3 Department of Gastroenterology and Hepatology, Shinshu University School of Medicine, 3-1-1 Asahi, Matsumoto, 390-8621 Japan; 4 Department of Biostatistics, Hyogo College of Medicine, 1-1, Mukogawa-cho, Nishinomiya, 663-8131 Japan; 5 Molecular Profiling Research Center for Drug Discovery, National Institute of Advanced Industrial Science and Technology, 2-4-7, Aomi, Koto-ku Tokyo, 135-0064 Japan

## Abstract

Fatty liver disease (FLD) increases the risk of diabetes, cardiovascular disease, and steatohepatitis, which leads to fibrosis, cirrhosis, and hepatocellular carcinoma. Thus, the early detection of FLD is necessary. We aimed to find a quantitative and feasible model for discriminating the FLD, based on plasma free amino acid (PFAA) profiles. We constructed models of the relationship between PFAA levels in 2,000 generally healthy Japanese subjects and the diagnosis of FLD by abdominal ultrasound scan by multiple logistic regression analysis with variable selection. The performance of these models for FLD discrimination was validated using an independent data set of 2,160 subjects. The generated PFAA-based model was able to identify FLD patients. The area under the receiver operating characteristic curve for the model was 0.83, which was higher than those of other existing liver function-associated markers ranging from 0.53 to 0.80. The value of the linear discriminant in the model yielded the adjusted odds ratio (with 95% confidence intervals) for a 1 standard deviation increase of 2.63 (2.14–3.25) in the multiple logistic regression analysis with known liver function-associated covariates. Interestingly, the linear discriminant values were significantly associated with the progression of FLD, and patients with nonalcoholic steatohepatitis also exhibited higher values.

## Introduction

Due to changes in lifestyles, the prevalence of fatty liver disease (FLD) has been consistently increasing worldwide especially in Asian regions^1–3^. Patients with FLD frequently do not exhibit signs or subjective symptoms until their illness becomes advanced, and such patients are at risk of developing steatohepatitis, fibrosis, cirrhosis, and hepatocellular carcinoma^2,4–6^. FLD also increases the risk of developing insulin resistance, diabetes, and cardiovascular diseases^7^. Thus, the early detection of FLD, which triggers earlier lifestyle modification, is necessary. FLD can be identified and diagnosed by abdominal ultrasound scans. However, the use of ultrasound as a diagnostic method is problematic because the inspection takes time and is unlikely to be conducted during a routine physical examination. Instead, subjects with suspected liver damage are initially screened by biochemical analyses, including the assessment of aspartate aminotransferase (AST) and alanine aminotransferase (ALT) serum level^8^. Because these enzymes are released from damaged hepatocytes into the blood following hepatocellular injury or death, they are not specific biomarkers of a fatty liver^8^.

In clinical settings, biomarkers generated from metabolomics are becoming one of the most important diagnostic criteria that can be objectively measured and evaluated as indicators of normal or pathological states, as well as a tool for assessing the outcome of therapeutic interventions. Focused-metabolomics, with well-managed sample collection, and accurate and reproducible measurements appear to be a realistic approach^9^. One of the traditional examples using a combination of plasma free amino acid (PFAA) profiles for diagnostic markers is Fischer’s ratio, which is the ratio of branched-chain amino acids (BCAAs: valine, leucine, isoleucine) to aromatic amino acids (AAAs: tyrosine, phenylalanine). Fischer’s ratio is used as a diagnostic marker for assessing liver metabolism, hepatic functional reserve, and the severity of liver dysfunction^10,11^. As liver dysfunction progresses, the plasma levels of BCAAs are decreased and AAAs are increased, respectively, thereby decreasing Fischer’s ratio.

Recent technological advances in focused-metabolomics have uncovered several metabolic signatures associated with FLD, including plasma amino acids^12,13^. However, to date, few quantitative studies have been performed in a large population, especially with respect to the optimal combination of PFAAs to evaluate the presence of FLD. In this study, we aimed to find a quantitative and feasible model for discriminating the FLD, based on PFAA profiles and confirmed the association with the progression of FLD.

## Results

### Construction of the robust PFAA- based fatty liver model (FLM)

We constructed a robust PFAA-only-based FLM using the data from 2,000 subjects to identify FLD patients. The performance of this model was confirmed in the independent validation data set of 2,160 subjects. The training data set consisted of 2,000 individuals (1,151 males and 849 females), 465 of whom were diagnosed as having FLD (Table 1). The validation data set consisted of 2,160 individuals (1,320 males and 840 females), 499 of whom were diagnosed as having FLD. Table 2 presents the concentrations of PFAAs. The levels of alanine (Ala), histidine (His), isoleucine (Ile), leucine (Leu), lysine (Lys), methionine (Met), ornithine (Orn), phenylalanine (Phe), proline (Pro), tryptophan (Trp), tyrosine (Tyr), and valine (Val) were significantly higher in patients with FLD, whereas the levels of citrulline (Cit), glycine (Gly), and serine (Ser) were lower in patients with FLD. In the FLM, which was chosen with regard to Akaike’s information criterion (AIC) and the likelihood ratio test, asparagine (Asn), Gly, Ala, Leu, Tyr, and arginine (Arg) were selected as explanatory variables. The amino acids which were elevated, reduced, and sustained in the patients with FLD were selected in this model. The resultant FLM was used for the following validation and characterization.

**Table 1:** Demographic and clinical characteristics of the healthy controls (HC) and the patients with fatty liver disease (FLD).

	Training data set		Validation data set	
Group	HC	FLD	HC	FLD
N	1535	465	1661	499
(Male, Female)	(784, 751)	(367, 98)	(932, 729)	(388, 111)
Age (years)	53.4 ± 11.1	53.7 ± 10.0	58.7 ± 10.9	57.8 ± 9.6
Body weight (kg)	58.9 ± 10.5	72.0 ± 11.9***	59.1 ± 10.8	70.5 ± 11.1***
BMI (kg/m^2^)	21.9 ± 2.7	26.0 ± 3.3***	22.0 ± 2.8	25.6 ± 3.1***
WC (cm)	80.2 ± 8.1	91.3 ± 8.0***	80.9 ± 8.5	90.8 ± 7.7***
HDL-C (mg/dL)	63.6 ± 14.7	51.3 ± 12.1***	63.9 ± 15.5	52.6 ± 11.4***
LDL-C (mg/dL)	122.9 ± 29.5	136.6 ± 32.0***	122.2 ± 29.1	131.1 ± 29.6***
Triglyceride (mg/dL)	96.9 ± 66.5	159.2 ± 87.8***	99.0 ± 73.4	159.8 ± 94.4***
Total protein (g/dL)	7.1 ± 0.4	7.3 ± 0.4***	7.2 ± 0.4	7.3 ± 0.4***
T-CHO (mg/dL)	205.3 ± 32.3	212.5 ± 34.4***	208.0 ± 31.5	210.7 ± 32.2
FPG (mg/dL)	94.7 ± 14.1	106.5 ± 20.7***	96.3 ± 15.7	107.4 ± 21.2***
HbA1c (%)	5.6 ± 0.5	5.9 ± 0.8***	5.7 ± 0.5	6.0 ± 0.7***
Insulin (μU/mL)	5.3 ± 3.0	9.8 ± 5.2***	5.5 ± 3.0	10.1 ± 5.5***
HOMA-IR	1.3 ± 1.1	2.6 ± 1.6***	1.3 ± 0.9	2.7 ± 1.7***
SBP (mmHg)	121.0 ± 18.2	132.3 ± 17.5***	120.4 ± 17.5	129.3 ± 16.0***
DBP (mmHg)	76.4 ± 11.0	83.3 ± 10.5***	75.9 ± 10.7	81.3 ± 10.0***
AST (U/I)	21.3 ± 8.6	26.8 ± 13.0***	20.6 ± 6.1	26.5 ± 16.1***
ALT (U/I)	20.0 ± 11.9	36.8 ± 22.6***	18.7 ± 11.8	33.2 ± 21.8***
LDH (U/I)	166.0 ± 29.9	176.9 ± 32.8***	174.2 ± 31.8	177.3 ± 30.0
ALP (U/I)	201.5 ± 61.7	217.3 ± 62.2***	202.9 ± 61.4	219.1 ± 60.2***
γ-GTP (U/I)	38.3 ± 43.3	64.0 ± 65.7***	36.3 ± 40.7	64.1 ± 137.7***
Fischer’s ratio	3.3 ± 0.5	3.4 ± 0.5***	3.3 ± 0.5	3.4 ± 0.5***
VFA (cm^2^)	107.4 ± 61.7	164.1 ± 51.9***	105.9 ± 55.3	176.9 ± 52.7***

Continuous variables are summarized as means ± standard deviations, and were compared between HC and FLD groups with the use of Welch’s t-test (*p < 0.05, **p < 0.01, and ***p < 0.001). In training data set, one WC value is missing in HC group and two WC values are missing in FLD group; therefore these individuals were not included. BMI: body mass index, WC: waist circumference, HDL-C: high-density lipoprotein cholesterol, LDL-C: low-density lipoprotein cholesterol, T-CHO: total cholesterol, FPG: Fasting plasma glucose, HbA1c: hemoglobin A1C, HOMA-IR: homeostasis model assessment of insulin resistance, SBP: systolic blood pressure, DBP: diastolic blood pressure, AST: aspartate aminotransferase, ALT: alanine aminotransferase, LDH: lactate dehydrogenase, ALP: alkaline phosphatase, γ-GTP: gamma-glutamyl transpeptidase, VFA: visceral fat area by computed tomography.

**Table 2:** The plasma free amino acid levels in the healthy controls (HC) and the patients with fatty liver disease (FLD).

	Training data set		Validation data set	
Group	HC	FLD	HC	FLD
N	1535	465	1661	499
Alanine	327.6 ± 70.4	386.0 ± 71.0***	327.3 ± 68.4	389.0 ± 68.0***
Arginine	90.3 ± 17.2	92.0 ± 15.7	89.1 ± 17.1	90.5 ± 16.0
Asparagine	45.5 ± 6.7	44.9 ± 6.5	45.1 ± 7.2	44.7 ± 6.5
Citrulline	31.0 ± 7.4	30.0 ± 6.8**	31.4 ± 7.3	30.0 ± 6.5***
Glutamine	572.3 ± 64.3	570.5 ± 64.3	559.7 ± 68.0	560.2 ± 64.5
Glycine	212.4 ± 49.8	186.2 ± 36.1***	212.2 ± 53.0	187.8 ± 37.4***
Histidine	79.7 ± 9.2	83.3 ± 10.3***	78.6 ± 9.2	82.2 ± 10.0***
Isoleucine	58.0 ± 13.4	71.6 ± 14.4***	57.7 ± 12.9	71.0 ± 14.2***
Leucine	115.1 ± 22.9	138.2 ± 23.1***	113.9 ± 21.9	136.2 ± 23.0***
Lysine	181.8 ± 30.2	196.1 ± 26.3***	183.9 ± 29.3	198.8 ± 28.8***
Methionine	25.1 ± 4.2	27.5 ± 4.4***	24.7 ± 4.1	26.8 ± 4.2***
Ornithine	49.5 ± 12.2	52.6 ± 10.6***	51.9 ± 12.9	56.2 ± 12.6***
Phenylalanine	56.7 ± 8.1	62.9 ± 10.0***	56.6 ± 8.0	61.8 ± 8.1***
Proline	129.4 ± 39.4	153.1 ± 41.7***	129.7 ± 40.1	153.2 ± 39.8***
Serine	112.0 ± 19.1	105.0 ± 16.6***	110.5 ± 18.5	107.0 ± 17.1***
Threonine	120.5 ± 25.0	121.1 ± 23.3	118.2 ± 24.4	121.5 ± 22.5**
Tryptophan	56.7 ± 8.9	62.5 ± 9.1***	55.6 ± 8.5	62.0 ± 8.8***
Tyrosine	61.0 ± 10.9	71.2 ± 11.1***	60.9 ± 10.5	70.8 ± 11.4***
Valine	210.8 ± 38.3	249.9 ± 38.5***	209.9 ± 37.5	246.9 ± 38.7***

Unit is μmol/L. Continuous variables are summarized as means ± standard deviations, and were compared between HC and FLD groups with the use of Welch’s *t*-test (*p < 0.05, **p < 0.01, and ***p < 0.001).

### Discriminant capability of the PFAA- based FLM

**Figure 1:**
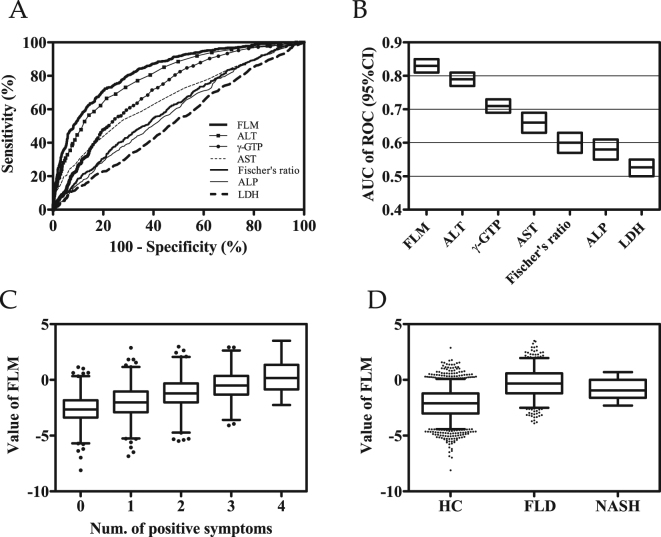
The discriminant capability of the plasma free amino acid-based model for fatty liver disease and association with metabolic dysfunction. (**A**) The ROC curves of the fatty liver model (FLM), ALT, γ-GTP, AST, Fischer’s ratio, ALP, and LDH for the discrimination between patients with fatty liver disease (FLD) and healthy subjects. (**B**) The values of the areas under the ROC curves (ROC_AUC) for discriminating the patients with FLD from healthy subjects. The top and bottom of the boxes indicate the 95% confidence interval of ROC_AUCs. (**C**) Relationship between the FLM value and the accumulation of risk factors associated with metabolic dysfunction. The FLM value was plotted against the number of risk factors associated with metabolic dysfunction (hypertension, hyperglycemia, hyperlipidemia, and visceral obesity). The box extends from the 25th to 75th percentiles. The line in the middle of the box is plotted at the median. The whiskers are drawn down to the 1st percentile and up to the 99th. Points below and above the whiskers are drawn as individual dots. The significant upward trend was illustrated by the Jonckheere-Terpstra trend test. (**D**) Box plot of FLM values for healthy controls (HC), patients with FLD, and patients with nonalcoholic steatohepatitis (NASH). ALT: alanine aminotransferase, γ-GTP: gamma-glutamyl transpeptidase, AST: aspartate aminotransferase, ALP: alkaline phosphatase, and LDH: lactate dehydrogenase.

The performance of the linear discriminant in the FLM and liver function-associated markers for discrimination between FLD patients and HCs was evaluated with the use of the receiver-operating-characteristic (ROC) curve and the estimate with 95% confidence interval (CI) of the area under the ROC curve (ROC_AUC). Between FLD patients and HCs, the ROC_AUC of the linear discriminant in the FLM was 0.84 (95% CI: 0.82, 0.86) in the training data set and 0.83 (95% CI: 0.81 to 0.85) in the validation data set (Fig. 1A,B). This ROC_AUC was statistically higher (Delong’s test: p < 0.001) than those of the following liver function-associated markers whose discriminating capabilities were as follows: the ROC_AUCs of gamma-glutamyl transpeptidase (γ-GTP), AST, Fischer’s ratio, alkaline phosphatase (ALP), and lactate dehydrogenase (LDH) were 0.72 (95% CI: 0.69 to 0.74), 0.66 (95% CI: 0.64 to 0.69), 0.60 (95% CI: 0.58 to 0.63), 0.58 (95% CI: 0.56 to 0.61), and 0.53 (95% CI: 0.50 to 0.56), respectively. The ROC_AUC of the linear discriminant in the FLM was also higher (Delong’s test: p < 0.01) than that of ALT whose ROC_AUC was 0.80 (95% CI: 0.78 to 0.82). Table 3 shows sensitivity, specificity, positive predictive value, negative predictive value, and efficiency of the linear discriminant in the FLM.

**Table 3:** Sensitivity, specificity, positive predictive value, negative predictive value, and efficiency of the fatty liver model (FLM) value in the validation data (N = 2,160).

Cutoff point	Sensitivity (%)	Specificity (%)	PPV (%)	NPV (%)	Efficiency (%)
First quintile	97	25	28	97	42
Second quintile	92	50	35	95	59
Third quintile	79	72	45	92	73
Forth quintile	54	90	62	87	82

PPV: positive predictive rate, NPV: negative predictive rate.

The odds ratios (ORs) for the FLM values for discriminating FLD are presented in Table 4. In the unadjusted models, the ORs associated with a 1 standard deviation (SD) increase in FLM value was 4.97 (95% CI: 4.23 to 5.84). The quintile analysis revealed a trend towards increased OR according to the FLM value (likelihood-ratio test: p < 0.001), and the ORs of top quintile group compared with the bottom quintile group were 48.79 (27.68 to 86.01). Adjusting for the variables affecting the likelihood of FLD (age, sex, AST, ALT, LDH, ALP, and γ-GTP), similar results were obtained, and the ORs associated with a 1 SD increase in FLM value was 4.96 (95% CI: 4.13 to 5.95). The ORs of the top quintile group compared with the bottom quintile group was 44.51 (21.40 to 92.60). These associations remained statistically significant after adjusting for following covariates: age, sex, AST, ALT, LDH, ALP, γ-GTP, weight, BMI, waist circumference, high-density lipoprotein cholesterol (HDL-C), low-density lipoprotein cholesterol (LDL-C), triglyceride (TG), total protein (TP), total cholesterol (T-CHO), Fasting plasma glucose (FPG), hemoglobin A1C (HbA1c), homeostasis model assessment of insulin resistance (HOMA-IR), systolic blood pressure (SBP), and diastolic blood pressure (DBP). The ORs associated with 1 SD increase in the FLM value was 2.63 (95% CI: 2.14 to 3.25). The quintile analysis revealed a trend towards increased OR according to the FLM value, and the ORs of the top quintile group compared with the bottom quintile group was 8.16 (95% CI: 3.49 to 19.07). We have also confirmed that similar performance was obtained using the training data set (Supplemental Figure 2). It suggests that the PFAA-based FLM was useful for identifying patients at high risk for FLD, even after adjusting for commonly accepted risk factors.

**Table 4:** Odds ratios (ORs) with 95% confidence intervals (CIs) for association between fatty liver disease and fatty liver model (FLM) value in the validation data (N = 2,160).

	Unadjusted	Adjusted^2^	Adjusted^3^
	OR (95%CI)	OR (95%CI)	OR (95%CI)
FLM value (transformed to *z*-score)			
Per SD^1^	**4.97 (4.23 to 5.84)**	**4.96 (4.13 to 5.95)**	**2.63 (2.14 to 3.25)**
p	<**0.001**	<**0.001**	<**0.001**
FLM value (classified into the quintile groups)			
First group	1.00 (reference)	1.00 (reference)	1.00 (reference)
Second group	1.91 (0.98 to 3.71)	1.86 (0.93 to 3.72)	1.31 (0.58 to 2.96)
Third group	**5.48 (3.03 to 9.91)**	**5.65 (2.96 to 10.76)**	1.58 (0.72 to 3.44)
Fourth group	**12.02 (6.79 to 21.30)**	**13.44 (7.01 to 25.78)**	**3.35 (1.61 to 6.99)**
Fifth group	**48.79 (27.68 to 86.01)**	**44.51 (21.40 to 92.60)**	**8.16 (3.49 to 19.07)**
p for trend	<**0.001**	<**0.001**	<**0.001**

^1^Per SD: standard deviation for FLM value.

^2^Adjusted for age, sex, AST, ALT, LDH, ALP, γ-GTP, and Fischer’s ratio.

^3^Adjusted for age, sex, AST, ALT, LDH, ALP, γ-GTP, Fischer’s ratio, weight, BMI, waist circumference, HDL-C, LDL-C, TG, TP, T-CHO, FPG, HbA1c, HOMA-IR, SBP, and DBP.

Significant odds ratios are highlighted in bold type. p for trend was calculated by likelihood-ratio test.

AST: aspartate aminotransferase, ALT: alanine aminotransferase, LDH: lactate dehydrogenase, ALP: alkaline phosphatase, γ-GTP: gamma-glutamyl transpeptidase, BMI: body mass index, HDL-C: high-density lipoprotein cholesterol, LDL-C: low-density lipoprotein cholesterol, TG: triglyceride, TP: total protein, T-CHO: total cholesterol, FPG: Fasting plasma glucose, HbA1c: hemoglobin A1C, HOMA-IR: homeostasis model assessment of insulin resistance, SBP: systolic blood pressure, DBP: diastolic blood pressure.

### The association of high FLM value with aggravating conditions of metabolic syndrome

The FLM value was significantly associated with the accumulation of metabolic syndrome-associated risk factors (Fig. 1C). The Jonckheere-Terpstra trend test demonstrated that the FLM value was elevated with the accumulation of risk factors (p < 0.001), suggesting that the FLM could identify subjects with risk factors to metabolic syndrome.

Furthermore, the subjects with nonalcoholic steatohepatitis (NASH) also exhibited higher FLM values compared with HCs (Fig. 1D). The FLM values for NASH patients were as high as those for patients with FLD, both of which were significantly higher than the values in HCs (Kruskal-Wallis test: p < 0.001, Dunn’s multiple comparisons test: p < 0.01 for HCs vs FLD patients; p < 0.05 for HCs vs NASH patients).

## Discussion

In this study, we used a focused metabolomic approach with well-managed sample collection and measurements with respect to reproducibility to generate a PFAA-based FLM. The FLM was able to discriminate between FLD patients and HCs even after adjusting for commonly accepted risk factors including age, sex, AST, ALT, LDH, ALP, γ-GTP, weight, BMI, waist circumference, HDL-C, LDL-C, TG, TP, T-CHO, FPG, HbA1c, HOMA-IR, SBP, and DBP (Table 4). The quintile analysis revealed a trend towards increased ORs according to the FLM value, and the OR of the top quintile compared with the bottom quintile was 8.16 (95% CI: 3.49 to 19.07) even after controlling for generally accepted risk factors. In this study, 465 of 2,000 subjects (23.3%) were classified as having FLD in the training data set, and 499 of 2,160 subjects (23.1%) were classified as having FLD in the validation data set (Table 1). Since it is reported that 26.2% of subjects who underwent comprehensive medical check-up were FLD by abdominal ultrasound scan^14^, the present population could be considered as a general Japanese population.

We recently reported the statistical approach called “AminoIndex technology”^9,15–17^ to compress multidimensional information from the PFAA profile into a single score to maximize the differences between the case and control population. It has been used to discriminate various physiological or disease states, including the progression of liver fibrosis in chronic hepatitis C patients^15^, visceral obesity and comorbidities^18–20^, and the identification of a risk for cardiovascular disease in diabetic patients^21^. In order for a biomarker to be successful in clinical settings, it must surpass conventional methods with respect to reliability and discriminative capability and/or should be more informative about disease progression^9^.

The ROC_AUC of the linear discriminant in the FLM for identifying patients with FLD was higher than that of other liver function-associated markers, demonstrating a competitive edge over existing markers (Fig. 1A,B). If the liver is injured, the liver cells release ALT, γ-GTP, and AST into the blood, thereby raising the plasma levels and signaling the liver damage. Thus, these enzymes are sensitive indicators of liver damage caused by different types of disease but are not specific indicators for liver fat accumulation. Fischer’s ratio, which is the molar ratio of BCAAs to AAAs, is important for assessing the clinical stage of liver disease^10,11^. Fischer’s ratio decreases as hepatic fibrosis or hepatic dysfunction progresses and thus reflects the degree of hepatic impairment, however, Fischer’s ratio does not decrease as liver fat accumulates. More specifically, the BCAA levels were elevated in subjects with FLD and NASH in this study, which is consistent with previous reports of the elevation of peripheral BCAAs^12,13^, whereas the BCAA levels decrease as the hepatic impairment progresses in the later stage of liver diseases. Thus the obtained FLM and Fischer’s ratio are completely different. The important next step is to examine the appropriate cutoff point of FLM value and to compare the accuracy of the model with existing markers. Although we calculated sensitivity, specificity, positive and negative predictive values, and efficiency in Table 3, we have to examine the clinical usefulness by comparing to existing markers with appropriate cutoff points in the next study.

The PFAA alterations observed in these FLD patients (Table 2) might have been caused by both metabolic changes due to FLD and dietary habits. However, studies so far have suggested that metabolic shifts, rather than dietary habits, play a more significant role in PFAA alterations. Many reports suggest that PFAA profiles are altered by visceral obesity^18–20^ and insulin resistance^19,22–25^, resulting in an elevations in plasma BCAAs, AAAs, and Ala and a decrease in Gly levels. Tai *et al*.^23^ previously examined PFAA profiles in association with insulin resistance and dietary habit in 263 non-obese Chinese and Asian-Indian men and demonstrated that although dietary protein intake markedly differed between ethnic groups, it did not affect the PFAA levels. Rather, PFAA levels were strongly associated with HOMA-IR values. Paradoxically, a higher dietary intake of BCAAs has been reported to be related to a lower prevalence of being overweight or obese^26^, lower insulin resistance^27^, and decreased risk of diabetes^28^.

The reason for the increase in blood BCAAs in patients with FLD is thought to be due to the lower uptake of BCAAs into the muscles caused by decreased insulin action and decreased utilization of amino acids in muscles^29,30^. Moreover, BCAAs are metabolized in visceral adipose tissues as well, and insulin resistance causes a decrease in the expression of BCAA-catabolizing enzymes in adipose tissue^29,31,32^. Intriguingly, the FLM value was significantly associated with the progression of FLD in this study, and the patients with NASH also exhibited higher FLM values (Fig. 1C,D). Greater elevation in FLM value was associated with higher likelihood for the co-occurrence of metabolic risk factors (visceral obesity, dysglycemia, high blood pressure, and dyslipidemia). Thus, the change in PFAA profiles which is reflected in FLM seems to be metabolic shifts caused by FLD and concomitant insulin resistance. The levels of Gly and Ser, two glucogenic amino acids, were lower in FLD patients. One possible reason for the reduction of Gly and Ser is an increased consumption of these amino acids by enhanced gluconeogenesis^33^ together with a decreased production by the enhancement of glyceroneogenesis. It has been reported that glucose production from both Gly and Ser in hepatocytes is increased in diabetic individuals, whereas this type of glucose production is low in healthy conditions. Although the levels of several other amino acids, such as Ala, and Cit, changed in FLD patients, the reasons are unclear. Thus, the mechanisms and physiological meaning underlying the PFAA alterations require further investigation.

Although we generated a PFAA-based FLM to identify FLD patients, there are two limitations in this study. The first limitation is lack of alcoholic intake data. The FLD comprises alcoholic and non-alcoholic FLD. Since the pathology between non-alcoholic vs alcoholic FLD are different, PFAA profiles might be different as well. Thus, the future studies to investigate whether the current FLM can evaluate both non-alcoholic and alcoholic FLD are wanted. The second limitation is population bias. Since this FLM was generated in Japanese population which has relatively high FLD ratio^14^, the performance of the FLM might be different in other populations.

In conclusion, our focused-metabolomic approach generated FLM, which conferred independent and differing contributions to increasing fatty liver risk relative to the currently known risk factors. Further longitudinal studies involving the sequential monitoring to examine the association between the FLM value and liver disease development, such as later NASH stages, fibrosis, cirrhosis, or hepatocellular carcinoma could be of importance for clarifying the physiological meaning of PFAA alterations.

## Methods

### Ethics

The study was conducted in accordance with the Declaration of Helsinki, and the protocol was approved by the Ethical Committees of Mitsui Memorial Hospital and the Ethical Committees of Shinshu University School of Medicine. All subjects gave their informed consent for inclusion before they participated in the study. All the data were analyzed anonymously throughout the study. The study was registered in the University Hospital Medical Information Network Clinical Trials Registry UMIN000015679.

### Control subjects and patients with FLD

The main inclusion criteria were as follows; generally healthy Japanese subjects with or without FLD who had undergone the Ningen Dock comprehensive medical check-up system^34^ in 2008 at the Center for Multiphasic Health Testing and Services, Mitsui Memorial Hospital in Tokyo, not taking antidiabetic medications regularly, not having serious health problems, and at least 20 years old (N = 4,160). The patients with hepatitis C or hepatitis B were excluded.

The individuals were divided into two data sets. The first 2,000 chronologically ordered individuals were included in the training data set, and the remaining 2,160 individuals were included in the validation data set. Both training and validation data sets were composed of fatty liver occurrence data (yes = 1, no = 0), PFAA profile data, and demographic and clinical characteristics’ data. The demographic and clinical characteristics of the patients are depicted in Table 1. Among all the data, there were 3 waist circumferences measures missing; therefore, these individuals were not included. Outliers were not excluded from the analyses, since they did not make a significant difference to the results.

### NASH patients

NASH patients (N = 10) who had undergone liver biopsy at the Shinshu University School of Medicine who have alcoholic intake data were recruited (Supplementary Table 1). Informed consent was obtained from each patient included in the study. For diagnosing NASH, liver biopsy specimens were obtained from segment 5 or 8 using 14-G needles and immediately fixed in 10% neutral formalin, embedded in paraffin, cut at 4-µm thickness, and stained with the hematoxylin and eosin or using the Azan–Mallory method. The average length of the samples was 15 ± 3 mm, and the average number of portal tracts found in each sample was 11 ± 4.

Histological findings were assessed by an independent experienced pathologist in a blinded fashion and scored according to the staging/grading system proposed by Kleiner *et al*.^35^. The nonalcoholic fatty liver disease histological activity score was calculated as the unweighted sum of the scores for steatosis (0–3), lobular inflammation (0–3), and ballooning (0–2). The histological diagnosis of steatohepatitis was made by the presence of macrovesicular steatosis, hepatocyte ballooning, and lobular inflammation.

### Analyses of biochemical variables and quantification of PFAAs

Blood samples were taken from the individuals after an overnight fast. FPG, and serum levels of T-CHO, HDL-C, LDL-C, TG, TP, and HbA1c were determined. Serum insulin levels were measured immunologically, and the HOMA-IR was calculated. The SBP and DBP were measured. Biochemical variables related to liver condition were measured, including AST, ALT, γ-GTP, ALP, and LDH. Liver fat content was examined by the ultrasound hepatic/renal ratio. The visceral fat area (VFA) was calculated from computed tomography images, and a 75 g oral glucose tolerance test was performed to obtain their 2-h post-challenge glucose levels for detecting the risk of diabetes in 865 subjects. The measurements of other variables were performed as previously described^19,20,36^.

For the amino acid analyses, blood samples (5 mL) were collected from forearm veins after overnight fasting into tubes containing disodium ethylenediaminetetraacetate and were immediately placed on ice. The plasma amino acid concentrations were measured by high-performance liquid chromatography–electrospray ionization mass spectrometry followed by precolumn derivatization as previously described^37–41^. The following 19 amino acids were measured: Ala, Arg, Asn, Cit, glutamine, Gly, His, Ile, Leu, Lys, Met, Orn, Phe, Pro, Ser, threonine, Trp, Tyr, and Val.

### Definition of metabolic syndrome related risk factors

According to the available biochemical data and computed tomography (CT) scanning data, the metabolic syndrome risk factors included hypertension, hyperglycemia, hyperlipidemia, and VFA were defined. The following criteria were used: hypertension, SBP equal to or higher than 140 mmHg or DBP equal to or higher than 90 mmHg; hyperglycemia, FPG equal to or higher than 110 mg/dL or blood glucose level equal to or higher than 140 mg/dL after oral glucose tolerance test; hyperlipidemia, LDL-C equal to or higher than 140 mg/dL or TG equal to or higher than 150 mg/dL; and visceral obesity, VFA by CT scan equal to or higher than 100 cm^2^. For VFA data, the number of individuals for whom the data were available was 865.

### Statistical analysis

#### Description and comparison of demographic and clinical characteristics

Demographic and clinical characteristics are presented as means with SDs, and were compared between the healthy controls (HCs) who had no apparent FLD and FLD patients with the use of Welch’s *t*-test.

### Construction of a robust PFAA-based FLM

To construct a robust PFAA-only-based FLM that discriminated between FLD patients and HCs, a multiple logistic regression model were fitted with leave-one-out cross-validation (LOOCV) to fatty liver occurrence data (response variable) and PFAA profile data (explanatory variables) in the training data set. All the possible combinations of 19 kinds of PFAAs were tried under the constraint that the maximum number of PFAAs was less than seven to avoid a potential over-fitting issue and to attain a parsimonious model. The LOOCV consisted of the following steps: (a) one sample was omitted sequentially one by one from the data set; (b) the logistic regression model was estimated using the data in which one sample was omitted; (c) based on the estimated model, the value of linear discriminant for the omitted sample and AIC were calculated; (d) steps (a) to (c) were repeated until all the linear discriminant values of the data set were obtained. Consequently, the top 100 best-fit models were picked up with regard to AIC.

To obtain the best-fit model that was less affected by age and gender, the model with minimum AIC value was chosen as the final FLM, under the constraint that no statistically significant effects of incorporating age and sex were confirmed with the use of the likelihood-ratio test. Also, each effect of the PFAAs included in the final model was assessed with the use of Wald’s test.

### Validation and clinical characterization of FLM

Using the validation data set and NASH patients’ data set, the values of the linear discriminant in the obtained FLM (FLM values) were calculated, and then the FLM was validated as follows: (1) The performance of the linear discriminant in the FLM and liver function-associated markers for discrimination between FLD patients and HCs was evaluated with the use of the ROC curve and the estimate with 95% CI of the ROC_AUC. The ROC_AUC was compared with the use of Delong’s test^42^. Sensitivity (=num. of true positives/num. of FLD patients), specificity (=num. of true negatives/num. of HCs), positive predictive values (PPV = num. of true positives/num. of positive subjects), negative predictive values (NPV = num. of true negatives/num. of negative subjects), and efficiency (=num. of true positives and true negatives/num. of total subjects) were also estimated with the use of prevalence rate at 23.1% because 499 of 2,160 subjects were classified as having FLD in this validation data set, according to the quintile of the distribution of the FLM values; (2) Relationship between fatty liver occurrence and linear discriminant was assessed with the use of the logistic regression model with/without adjustment of demographic variables and metabolic syndrome related variables, where the FLM values were transformed to z-score and classified into five quintile groups. The ORs are presented with their 95% CIs and p values. Trend towards increased ORs among the five quintile groups was confirmed with the use of likelihood-ratio test; (3) Association of trend towards higher FLM values with the cumulative number of metabolic syndrome-related risk factors (summed up from 0 to 4 in each subject) was assessed with the use of the Jonckheere–Terpstra test; (4) Difference in the distribution of the FLM value among HCs, FLD patients, and NASH patients was assessed with the use of the Kruskal-Wallis test, followed by Dunn’s multiple pairwise comparison test.

### Software

MATLAB R2015a (The MathWorks, Natick, MA, USA) was used for all statistical analyses. GraphPad Prism 5 (GraphPad Software, La Jolla, CA, USA) was used for the ROC analysis. All probability values are two-sided, and value of p < 0.05 was considered statistically significant.

## Electronic supplementary material

Supplementary Tables
