# Supplementary material for: Plasma amino acid profile associated with fatty liver disease and co-occurrence of metabolic risk factors
Source: Sci Rep. 2017 Nov 3;7:14485. doi: 10.1038/s41598-017-14974-w (PMC5670226; doi:10.1038/s41598-017-14974-w)
Supplement: Supplementary file 1 — Supplementary Tables [file 41598_2017_14974_MOESM1_ESM.pdf]

Title: Plasma amino acid profile associated with fatty liver disease and co-occurrence of metabolic risk factors

Authors: Minoru Yamakado, Takayuki Tanaka, Kenji Nagao, Akira Imaizumi, Michiharu Komatsu, Takashi Daimon, Hiroshi Miyano, Mizuki Tani, Akiko Toda, Hiroshi Yamamoto, Katsuhisa Horimoto, Yuko Ishizaka

Supplementary Table 1

Clinical characteristics of patients with nonalcoholic steatohepatitis (NASH)

	NASH
N	10
(Male, Female)	(7, 3)
Age (years)	56.4±12.3
Body weight (kg)	66.1±12.3
Body mass index (kg/m^2)	26.6±4.3
HDL-C (mg/dL)	55.1±11.4
LDL-C (mg/dL)	142.2±40.5
Triglyceride (mg/dL)	142.5±54.8
Total protein (g/dL)	7.6±0.4
T-CHO (mg/dL)	219.6±39.7
FPG (mg/dL)	123.3±30.9
HbA1c (%)	6.2±0.9
HOMA-IR	4.3±3.1
SBP (mmHg)	125±14.6
DBP (mmHg)	75.3±8.7
AST (U/I)	66.3±40.0
ALT (U/I)	107.4±66.2
LDH (U/I)	233.7±48.9
ALP (U/I)	305.9±114.8
γ-GTP (U/I)	88.8±56.2
Fischer's ratio	3.4±0.7

Values are summarized as means ± standard deviations. HDL-C: high-density lipoprotein cholesterol, LDL-C: low-density lipoprotein cholesterol, T-CHO: total cholesterol, FPG: Fasting plasma glucose, HbA1c: hemoglobin A1C, HOMA-IR: homeostasis model assessment of insulin resistance, SBP: systolic blood pressure, DBP: diastolic blood pressure, AST: aspartate aminotransferase, ALT: alanine aminotransferase, LDH: lactate dehydrogenase, ALP: alkaline phosphatase, γ-GTP: gamma-glutamyl transpeptidase.

Supplementary Table 2

Odds ratios (ORs) with 95% confidence intervals (CIs) for association between fatty liver disease and fatty liver model (FLM) value in the training data (N=1,997)

	Unadjusted OR (95%CI)	Adjusted [2] OR (95%CI)	Adjusted [3] OR (95%CI)
FLM value (transformed to *z*-score)			
Per SD [1]	**4.88 (4.14 to 5.74)**	**3.98 (3.28 to 4.83)**	**2.30 (1.84 to 2.88)**
P	**<0.001**	**<0.001**	**<0.001**
FLM value (classified into the quintile groups)			
First group	1.00 (reference)	1.00 (reference)	1.00 (reference)
Second group	**3.92 (1.68 to 9.15)**	**3.82 (1.61 to 9.07)**	2.36 (0.96 to 5.77)
Third group	**9.55 (4.30 to 21.20)**	**7.88 (3.44 to 18.07)**	**3.81 (1.62 to 8.97)**
Fourth group	**25.11 (11.55 to 54.61)**	**18.63 (8.18 to 42.41)**	**6.24 (2.66 to 14.66)**
Fifth group	**91.37 (42.14 to 198.11)**	**53.62 (23.32 to 123.27)**	**11.75 (4.86 to 28.37)**
P for trend	**<0.001**	**<0.001**	**<0.001**

For calculating odds ratios, three individuals who have missing data (waist circumference data) were excluded from the analyses since adjustment cannot be done.

[1]Per SD: standard deviation for FLM value.

[2]Adjusted for age, sex, AST, ALT, LDH, ALP, γ-GTP, and Fischer's ratio.

[3]Adjusted for age, sex, AST, ALT, LDH, ALP, γ-GTP, Fischer's ratio, weight, BMI, waist circumference, HDL-C, LDL-C, TG, TP, T-CHO, FPG, HbA1c, HOMA-IR, SBP, and DBP.

Significant odds ratios are highlighted in bold type. P for trend was calculated by likelihood-ratio test.

AST: aspartate aminotransferase, ALT: alanine aminotransferase, LDH: lactate dehydrogenase, ALP: alkaline phosphatase, γ-GTP: gamma-glutamyl transpeptidase,

BMI: body mass index, HDL-C: high-density lipoprotein cholesterol, LDL-C: low-density lipoprotein cholesterol, TG: triglyceride, TP: total protein, T-CHO: total cholesterol, FPG: Fasting plasma glucose, HbA1c: hemoglobin A1C, HOMA-IR: homeostasis model assessment of insulin resistance, SBP: systolic blood pressure, DBP: diastolic blood pressure.
